# Transformative adaptation through nature-based solutions: a comparative case study analysis in China, Italy, and Germany

**DOI:** 10.1007/s10113-023-02066-7

**Published:** 2023-05-02

**Authors:** Anna Scolobig, JoAnne Linnerooth-Bayer, Mark Pelling, Juliette G. C. Martin, Teresa M. Deubelli, Wei Liu, Amy Oen

**Affiliations:** 1 International Institute for Applied Systems Analysis, Schlossplatz 1, 2361 Laxenburg, Austria; 2 University of Geneva, Geneva, Switzerland; 3 University College London, London, UK; 4 Norwegian Geotechnical Institute, Oslo, Norway

**Keywords:** Transformative adaptation, Nature-based solutions, Polycentric governance, Climate adaptation policy, Disaster risk reduction, Planning

## Abstract

This paper explores how claims for transformative adaptation toward more equitable and sustainable societies can be assessed. We build on a theoretical framework describing transformative adaptation as it manifests across four core elements of the public-sector adaptation lifecycle: vision, planning, institutional frameworks, and interventions. For each element, we identify characteristics that can help track adaptation as transformative. Our purpose is to identify how governance systems can constrain or support transformative choices and thus enable targeted interventions. We demonstrate and test the usefulness of the framework with reference to three government-led adaptation projects of nature-based solutions (NBS): river restoration (Germany), forest conservation (China), and landslide risk reduction (Italy). Building on a desktop study and open-ended interviews, our analysis adds evidence to the view that transformation is not an abrupt system change, but a dynamic complex process that evolves over time. While each of the NBS cases fails to fulfill all the transformation characteristics, there are important transformative elements in their visions, planning, and interventions. There is a deficit, however, in the transformation of institutional frameworks. The cases show institutional commonalities in multi-scale and cross-sectoral (polycentric) collaboration as well as innovative processes for inclusive stakeholder engagement; yet, these arrangements are ad hoc, short-term, dependent on local champions, and lacking the permanency needed for upscaling. For the public sector, this result highlights the potential for establishing cross-competing priorities among agencies, cross-sectoral formal mechanisms, new dedicated institutions, and programmatic and regulatory mainstreaming.

**Supplementary Information:**

The online version contains supplementary material available at 10.1007/s10113-023-02066-7.

## Introduction

A global discussion is taking place on the urgent need for transformative adaptation motivated not only by the impacts of climate change but also by biodiversity loss, soil and water pollution, and other planetary risks, reinforced by accelerating socioeconomic inequalities and exacerbated by COVID-19. Indeed, over the last decade, transformative adaptation to climate change has become part of the policy discourse. Claims for transformation have been made across many policy domains (Frantzeskaki et al. 2020). While multiple understandings of transformative adaptation persist, including the view that transformation is an extension of, or stands in opposition to, incremental adaptation, core elements are emerging from climate adaptation practice.

A wide consensus is emerging in the global community that the ultimate goal of transformative adaptation is to support more equitable and sustainable societies at the international, national, and local scales. In this context, transformative change has generally been defined as change that challenges the status quo, namely, alterations in a system’s fundamental nature, state, structure, or function (Smith & Stirling 2010; O’Brien 2011; Béné et al. 2012). The IPCC Special Report on Warming Above 1.5 Degrees (2018b, p. 559) extended this definition to encompass “a system-wide change that requires more than technological change through consideration of social and economic factors that, with technology, can bring about rapid change at scale.”

Social transformation is a key component of transformative adaptation. It is a profound and often deliberate shift initiated by communities toward sustainability, facilitated by changes in individual and collective values and behaviors, and a fairer balance of political, cultural, and institutional power in society (IPCC 2018b, p.558). Transformative adaptation, if it contributes to the UN Sustainable Development Goals (SDGs) by creating inclusive, equitable, and resilient sustainable development opportunities that “leave no-one behind,” brings a normative orientation that takes transformative adaptation above and beyond a simple description of fundamental change (Folke et al. 2010; Pelling 2010; Pelling et al. 2015; Faldi and Macchi 2017; Bosomworth 2018).

Moving from definitions to practice, claims of transformative adaptation have been documented across many sectors and contexts, including agriculture, ecosystems, cities, management of deltas and polders, mineral extraction, water conservation/management, and area planning by relocation (Kates et al. 2012; Vermeulen et al. 2018; Chu et al. 2019; Deubelli and Mechler 2020; Zografos et al. 2020). These studies illustrate not only how transformative adaptation works but also the enthusiasm and willingness of some researchers, policymakers, and practitioners to claim that interventions and outcomes have transformative *status*.

Although there is consensus on the aims of transformative adaptation to promote sustainable and equitable societies, and there are multiple successful examples, the question of how to achieve transformation is still evolving. Evidence is thin regarding the types of actions needed within economic, technological, and governance systems and how competing aspirations and values can be balanced in determining what to transform and what a transformed end-state might look like. Moreover, although there are multiple understandings of transformative adaptation, not all fit the definition of fundamental change toward more equitable, resilient, and sustainable futures (Folke et al. 2010; Pelling 2010; Faldi and Macchi 2017; Bosomworth 2018). This is a core aspect of debates on the intention and impacts of claimed transformations, including those defined by their relationships to incremental adaptation (Mustelin and Handmer 2013).

To address this question, recent literature has sought to distinguish the characteristics and modalities of transformative adaptation. For example, Watkiss and Cimato (2020) discern three defining characteristics of transformative interventions: the size or scale of an intervention, its temporality, and its domain. Yet, analysis is constrained by the absence of a common agreement on the defining criteria used in making such a claim, and the level of change that may qualify as “transformational” often remains relative and contextual (Rickards and Howden 2012; Termeer et al. 2017; Deubelli and Mechler 2020).

The aim of this paper is to demonstrate an assessment framework for evaluating the transformative potential of government-led and government-financed adaptation projects. While claims for transformative adaptation can take place across diverse market and non-state activities, for example, innovative insurance products that reward disaster risk mitigation or inclusive educational programs that provide disaster resilience, we limit the scope of our analysis to government-financed projects that promote climate adaptation. We further limit the analysis to public administration by examining the processes that have led to adaptation decisions once a budget was available. In other words, we do not examine the political processes that resulted in project prioritization and financing. This does not mean that market and non-state actors are not involved; to the contrary, we show that these actors are critically important in enabling publicly financed adaptation investments (see also Dodman et al. 2022). 

Moreover, Our point of departure is the view that transformation is not a “one-off” system change, but a complex and nuanced set of actions involving diverse actors that evolve over time.

**Fig. 1:**
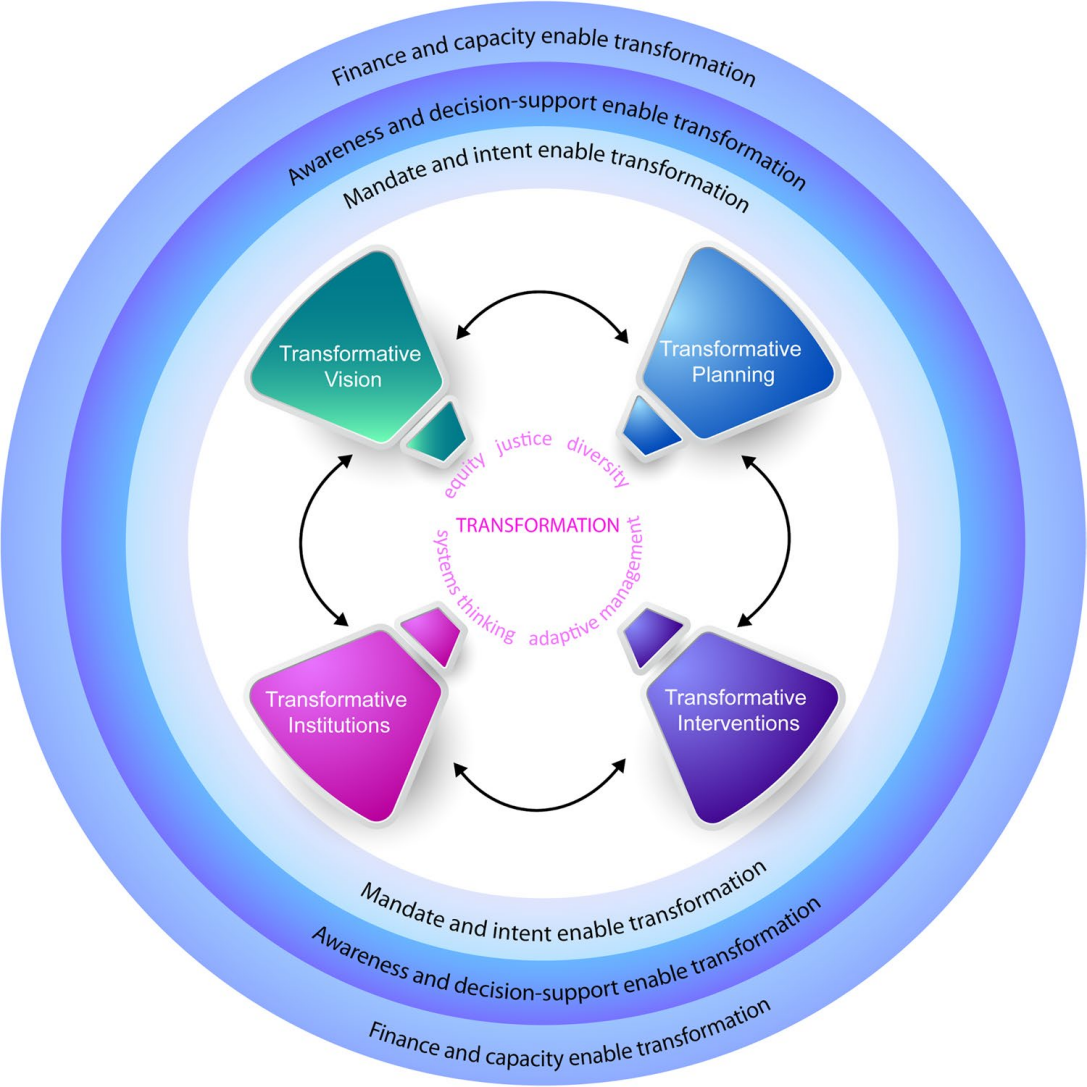
A framework for transformative adaptation in government projects

Specifically, we emphasize four key elements of government-financed adaptation investments: vision, planning, interventions, and institutions that, together, can be employed to contribute to delivering transformation. Each element describes an action space for decision-making where transformative outcomes can be delivered. Acknowledging that transformative adaptation can be independent, the elements are not presented as a cycle (see Fig. 1). No preceding stage is required for transformative action to arise within an element, and transformative action might only be found in one element; indeed, transformative outcomes might not be realized at the time of observation. This said, the most coherent and purposeful transformative outcomes will likely pass intentionally through at least one round of vision, planning, intervention, and institutionalization (including organizational learning associated with monitoring and learning with scope to feedback into policy). Rather than focusing on the temporal dimension of the policy cycle (e.g., agenda setting, decision-making, implementation, and evaluation Cairney, 2012; Knill and Touson, 2012) or the steps needed to implement adaptation solutions, our framework highlights the key elements and characteristics of transformation within the context of administrative governance. The elements allow a targeted analysis of transformation processes. This opens scope for case specific process indicators to be developed from the established and complementary adaptation literature that tends to focus on outputs (Deubelli and Mechler 2020) and overlook transformative action, and its frustration, within policy-practice processes (Fedele et al. 2019a, b). A systematic understanding of these processes is key for fostering further transformative actions and outcomes.

The test case for the framework is adaptation through nature-based solutions (NBS), which are defined by the United Nation Environment Assembly as actions taken “to protect, conserve, restore, sustainably use and manage natural or modified terrestrial, freshwater, coastal and marine ecosystems, which address social, economic and environmental challenges effectively and adaptively, while simultaneously providing human well-being, ecosystem services and resilience and biodiversity benefits.” (UNEA 2022; for an overview of NBS definitions see White House 2022).

As such, NBS are well positioned to meet the goals of sustainable and equitable societies. Indeed, investing in nature has proven to be a promising strategy for climate adaptation, improving ecosystem-based disaster risk reduction, increasing social and ecological resilience, protecting ecosystems, and improving livelihoods through the maintenance, restoration, enhancement, and sustainable use of ecosystems and their services (de Jesús Arce-Mojica et al. 2019; Palomo et al. 2021). Nature-based solutions contribute to different aspects of adaptation. In particular, there is robust evidence showing they can reduce direct exposure to climate change impacts. For example, restoring native ecosystems can promote healthy soil and vegetation that reduce the risks of floods, droughts, and landslides by increasing infiltration and storage of water, stabilizing slopes and shores, and attenuating wave energy (Seddon 2022).

Promoting NBS for adaptation is also becoming a key objective of climate policy. For example, in 2022, NBS were for the first time included in the Conference of the Parties (COP 27) decision text that “encourages Parties to consider, as appropriate, nature-based solutions or ecosystem-based approaches, taking into consideration United Nations Environment Assembly resolution 5/5,31 for their mitigation and adaptation action while ensuring relevant social and environmental safeguards” (UNEA 2022). NBS are also included in the new EU Strategy on Adaptation to Climate Change, in which NBS are considered essential for increasing climate resilience and sustaining healthy water, oceans, and soils (EC 2021). Moreover, at least 66% of the signatories to the Paris Agreement include NBS in some form to help achieve their climate change mitigation or adaptation goals (Scolobig et al. 2020). To support effective NBS implementation, several global standards and guidelines have been developed and are presently used to support national policy developments in different countries (e.g., IUCN 2020).

In this context, we identified a policy relevant research gap in the literature. NBS (including ecosystem-based adaptation) have achieved rapid prominence worldwide with increasing funding support and are often associated with claims for significant co-benefits for social justice (livelihood, health, and wellbeing) and ecology. In this way, NBS are explicitly or implicitly presented as enabling of transformative adaptation. However, it is not clear if and how NBS can lead to transformative adaptation. To address this gap, we demonstrate the usefulness of a framework for evaluating the transformative potential of government-led adaptation projects at the local or subnational government level by examining three empirical cases of NBS implementation aimed at disaster risk reduction and climate change adaptation in China, Germany, and Italy. More precisely, we critically discuss if and how the vision, planning, interventions, and institutional frameworks implemented in the three case studies can be considered as transformative. We do this on the basis of a comparative case study analysis consisting of an in-depth desktop study and 47 semi-structured interviews with stakeholders involved in the NBS implementation process, including public administrators at all scales, non-governmental organizations, households, and businesses (Martin et al. 2019). After presenting the results of our analysis, we discuss them in light of recent literature, and we reflect on the gaps, emerging limitations of the framework and future research directions.

## Background

Many forms of adaptation — whether carried out by government, businesses, civil society organizations, or individuals — respond to the interrelated challenges of development and climate resilience, and in so doing promote the dual goals of sustainability and social equity. The rapidly emerging experience with transformative adaptation by governments indicates that a large proportion of the tools of everyday project planning, financing, negotiation, and political leadership can be deployed. Judgments on the appropriateness of specific adaptation options balance immediate need (e.g., adapting housing design) with medium-term planning (e.g., urban zoning) and longer-term investments (e.g., research and development) (Watkiss and Cimato 2020). Adaptation policy options need to be viewed in their social and political context. Spontaneous and planned projects are usually also part of policy/sector adaptation processes, and these, in turn, contribute to national pathways for adaptation.

As we argue in this paper, the delivery of transformation for publicly financed investments may occur as part of an established adaptation project. The difference lies primarily in intention and associated preferences for creating the vision, planning procedures, interventions, and governance structures for institutionalizing transformative gains. As shown in Fig. 1, our framework sets out these four key elements, which together can be used to better understand transformative adaptation in government-financed projects. This moves the analysis from a zero-sum assessment of actions as transformative (or not) toward a more nuanced understanding of those elements in a policy or project cycle that are more-or-less transformative. This, in turn, can highlight where specific decision-making and implementing systems constrain transformative choices and so enable targeted interventions to address blockages and open systems up to the full range of adaptation possibilities.

An important feature of the framework is that while transformation can arise from a “one-off” event, it is more likely to be an emergent property, the result of interaction between multiple policy processes and interventions involving diverse stakeholders over time (Nalau and Handmer 2015). Indeed, transformative adaptation can be — and often is — a complex process; it may occur over long timeframes and it may involve events that extend over the lifetime of a project or policy. Transformative processes viewed in this way might include setbacks and incremental steps toward a transformative goal, as well as single acts of transformative change. The framework also recognizes that the extent to which a public investment (or intended investment) might be transformative is shaped by several factors. These include as follows: the agent’s mandate and intent; awareness of adaptive choices and consequences determined by a decision-support environment; and available capacities, including finance and policies that can be drawn upon for planning and implementation (Deubelli and Mechler 2020; see also Fig. 1). Moreover, government-led processes can be rife with stakeholder conflict rooted in both stakeholder interests and deeply held values or worldviews (Hajer 1997; Dryzek 2001; Thompson and Rayner 1998).

Seen through the lens of “transformation,” our framework differs conceptually not only from the typical policy cycle as described above but also from the adaptation policy cycle (e.g., risk assessment, identification of policy options, option assessment, implementation, and monitoring; EEA 2015) because it sets out the core elements of transformative adaptation. Adaptation policy cycles typically describe what steps need to be taken to implement adaptation solutions. Thus, the temporal dimension plays a critical role, unlike the framework that we propose, which highlights the key elements and characteristics of transformative adaptation. Yet, the temporal dimension is clearly embedded within each of the elements and within the ways they are delivered (e.g., transformative planning). Moreover, our framework differentiates itself from the policy cycle by recognizing that policy typically emerges from an implicit or explicit negotiation among competing advocacy groups or coalitions (Jenkins-Smith and Sabatier 1994), which calls for an understanding of the complex subsystem interactions among government officials, private actors, experts, and civil society.

If we are to characterize transformative adaptation according to this framework, the salient characteristics of transformative visions, plans, interventions, and institutional frameworks (i.e., governance structures for institutionalizing transformative gains) must be identified. For this, we make use of recent and systematic literature reviews that identify criteria that can be associated with transformative adaptation. Specifically, Deubelli and Mechler (2020) and Watkiss and Cimato (2020) have mapped and categorized attributes, criteria, and characteristics for transformational adaptation that are set out in the literature (see, e.g., Mustelin and Handmer 2013; Londsale et al. 2015; Tàbara et al. 2018; Fazey et al. 2018; Fedele et al. 2019a, b; Pal et al. 2019; Zografos et al. 2020).

Building on this literature, we assign “transformative characteristics” to each of the framework elements shown in Fig. 1. The purpose is not to reinvent the characteristics of transformative adaptation, but rather to organize them into four key elements. In summary, this framework can assist transformation and help to understand if and when it has occurred. It can be used to support decisions on adaptation options, inspire improvement, and foster learning processes (Fazey et al. 2018).

Table 1 lists the framework characteristics along with their description, examples, and references. Importantly, the table suggests common and consistent characteristics for transformation that emerge and on which it is important to build. We argue that transformative adaptation is most likely to result from initiatives that display these characteristics, or at least some of them (see also Pal et al. 2019).

**Table 1:** Characteristics of transformative vision, planning, interventions, and institutional frameworks

Framework elements	Characteristic	Description	Example	Examples of references
Transformative vision	Systemic	A transformative vision is comprehensive and systemic; it addresses needs and changes beyond component challenges to cover wider relational issues. It includes radically new concepts, notions, and opportunities	“Making space for the river,” a Dutch concept that encompasses comprehensive shifts in urban planning and flood protection to enhance ecosystem function and minimize long-term vulnerability	Fazey et al. (2018) Tabara et al. (2018) Pal et al. (2019) Fedele et al. (2019a, b)
	Path shifting	A transformative vision breaks from the status quo to embrace fundamental alterations in natural and social systems	The proposed European Commission’s taxonomy on nature-negative investments, which can form the basis for divestments from unsustainable projects	Fedele et al. (2019a, b) Zografos et al. (2020) Vysna et al. (2021)
	Mobilizes advocacy coalitions	A transformative vision mobilizes human resources, e.g., advocacy coalitions and/or local champions	Nature-based solutions implementation strongly advocated by NGOs champions or advocacy groups, e.g., in Genk (Belgium)	Martin et al. (2019), Frantzeskaki et al. (2020)
	Addresses root causes or drivers	A transformative vision addresses deep causal relationships between risk factors, e.g., the drivers of climate risks and vulnerabilities	In Genova (Italy), root causes of risk that were addressed by dealing with legal conflicts hindering the implementation of disaster risk reduction measures	Pelling (2010) Fraser et al. (2016), Zografos et al. (2020) Scolobig (2017)
Transformative planning	Inclusive	Transformative planning involves all interested and affected stakeholders and takes account of the range of diverse views	Sediment management strategy along the banks of the Kosi River, Bihar, India, developed in consultation with stakeholders	Martin et al. (2019) Pal et al. (2019)
	Co-production	Transformative planning produces policy options and solutions jointly with policy makers, experts, and stakeholders. It recognizes expert and local knowledge	Co-design of climate risk reduction plan involving residents living in risky areas and other stakeholders	Tabara et al. (2018), Watkiss and Cimato (2020)
	Equitable	Transformative planning prioritizes vulnerable groups and includes an assessment of equity and distributional issues	Implementation of measures that take into account differential social vulnerabilities	Tabara et al. (2018)
	Based on open data systems	Transformative planning is based on transparent and open access data collection and analysis	Data used in the planning process made available to all stakeholders involved, e.g., upgrading organizational capacities to collect and analyze climate statistics in Nepal	Pal et al. (2019)
Transformative interventions	Scalable	Transformative interventions are scalable to generate large scale or systemic impact	Successful pilot interventions embedded in a national policy	Moore et al. (2014) Pal et al. (2019)
	Sustainable in the long term	Transformative interventions deliver long-term economic, social, and environmental benefits after direct implementation support ends	Nature-based solutions for disaster risk reduction provide multiple long-term benefits (e.g., climate mitigation, wellbeing, and health-related effects)	Fazey et al. (2018) Fedele et al. (2019a, b) Pal et al. (2019) Chan et al. (2020)
	Future-oriented	Transformative interventions focus on long-term change and acknowledge uncertainty	People-centered early warning systems that take into account the changing patterns of climate extremes	Mustelin and Handmer (2013)
Transformative institutional frameworks	Polycentric	Transformative institutional frameworks recognize the value of diffusing governance centers through the revision of mandates, responsibilities, and organizational structures	Establishment of multi-sectoral or multi-scale governance structures (e.g., cross-sectoral committees) that will continue to deliver benefits beyond the intervention	Fedele et al. (2019a, b) Martin et al. (2019) Zografos et al. (2020)
	Promoting social justice	Institutional frameworks are transformative if they address power imbalances and social injustice	Adaptation finance that targets those most vulnerable to climate impacts	Mustelin and Handmer (2013) Chan et al. (2020)
	Catalytic	Institutions are transformative if they catalyze changes within structures that are beyond their direct mandate or reach	Multiple agencies use of a tool, model, or framework developed by one initiative, delivering co-benefits in other areas, and new feedback and accountability mechanisms	Fazey et al. (2018) Pal et al. (2019)

## Methodology

This analysis examines three case studies of government-financed projects that promoted climate adaptation through the implementation of nature-based solutions (NBS) for disaster risk reduction: the Isar River restoration in Munich, Germany; landslide mitigation in Nocera Inferiore, Italy; and reforestation in Wolong, China. The cases were chosen because of their diversity (hazard, country, policy context, stakeholder conflicts) and because each involved a lengthy and well-documented policy implementation process. Additionally, the three cases represent widely recognized, successful, and — in the view of stakeholders — innovative NBS implementation. Since there are few in-depth comparative and temporal studies of NBS implementation across different countries, the cases provide a unique opportunity to understand the complexity of real-world contexts (Flyvbjerg 2006; Frantzeskaki et al. 2020).

The case study methodology included as follows: (i) a desk-based review focused on the NBS projects; (ii) targeted open-ended interviews (telephone and face-to-face) with key informants involved in or affected by the planning and implementation of the projects; and (iii) participant observation during fieldwork in the case studies. The desk-based review included grey literature, scientific publications, reports, newspapers, and websites providing relevant information. The review, in turn, served as a preliminary identification of relevant stakeholders for the interviews. Interviewees were also identified through expert consultation and snowball sampling. The interview protocol included questions concerning the NBS project lifecycle, e.g., advocacy coalitions, the key enablers, and barriers to NBS implementation as well as essential characteristics of the vision, planning, interventions, and institutional frameworks. Our purpose was to identify the transformative characteristics of the NBS processes and outcomes, as set out in Fig. 1, drawing on the detailed data from the three NBS cases. Case studies are analyzed to draw conclusions and cross-case insights that inform existing theory and evidence on transformative adaptation (Yin 2014; Martin et al. 2021). We employed the strategy of selecting diverse cases across different countries in order to represent a larger variety of governance contexts in the analysis (Gerring and Cojocaru (2016) and George & Bennett (2005).

The case study data included 47 interview transcripts (see Annex 1 for a list of interviewees and interview details). Interviewees were selected through expert consultation and snowball sampling. Of these, 15 were conducted for the Isar case, 21 for the Nocera case, and 11 for the Wolong case. For each case, interviewees represented different sectors and administrative levels, as well as non-governmental entities involved in the design, planning, or implementation of the NBS project. Interview data were analyzed using qualitative content analysis (Bryman 2012; Yin 2014; Mayring 2014; Corbin et al. 2015) taking into account the national data protection laws in the different countries in the study. Interview data are available in original language upon request.

## Case studies

The three selected NBS projects focus, respectively, on (i) mitigating flood risk through the restoration of the Isar River in Munich, Germany; (ii) halting deforestation and encouraging afforestation as measures to reduce flood/landslide risk in the Wolong Nature Reserve, China; and (iii) reducing landslide risk and improving forest management with natural measures in Nocera Inferiore, Italy. The main characteristics of each case are summarized in Table 2 (for a detailed description of each case, see Martin et al. 2019).

**Table 2:** Selected characteristics of the Isar, Wolong, and Nocera cases

	Isar-Plan (2000–2011)	Wolong Nature Reserve (2000–present)	Nocera Inferiore (2015–2019)
Location	Munich, Germany	Sichuan Province, China	Campania, Italy
Risk	Flood	Flood and landslide	Landslide
NBS project	Riverbed restoration and creation of a green/blue recreational space	Forest management concession contractual system through the Natural Forest Conservation Program	Maintenance and remediation of the mountain slope, channel lining, and provision of vegetated and stone gabions
Main co-benefits	Flood risk reduction, ecological restoration, recreation	Afforestation, landslide and flood risk reduction, biodiversity conservation, socioeconomic development, carbon sequestration	Landslide risk reduction, forest management recreation, environmental awareness
Approximate cost	€35 million	€1 million/year	€637,000

Source: based on Martin et al. (2021)

Short descriptions of the three cases are provided below. In the “Emergent characteristics of transformative adaptation” section, we examine the case details on stakeholder visions leading to the NBS, the participatory planning process, the interventions for the NBS and responsible institutions, and the question as to which of these characteristics can be considered transformative.

### Isar case

In 2000–2011, an 8-km-long stretch of the Isar river in Munich (Germany) was restored using a hybrid of green and grey measures, referred to as the Isar Plan (Wasserwirtschaftsamt München and Landeshauptstadt München 2011). The measures implemented included the widening of the riverbed, increasing the water levels, the addition of natural material to enhance the riverine habitat quality and biodiversity, and the reinforcement of existing flood levees with underground steel beams to preserve vegetation. The Isar Plan was jointly financed and implemented by the State of Bavaria and the City of Munich and is widely acclaimed for having successfully turned a formerly concrete and unsafe riverbank into a green/blue recreational space, which became an emblem of the city (Binder 2010; Sartori 2012; Düchs 2014). The aims of the project were threefold: flood protection, environmental restoration (both of these fulfilling the Munich Water Agency’s main mandates), and creating an urban recreational space (fulfilling the City of Munich’s mandate as well as the demands of local councils and Munich’s inhabitants). A decade before the start of the project, environmental groups succeeded in claiming increased residual water for the Isar from the *Mühltal* hydropower plant for which the concession was expiring. Having won this battle, these same stakeholders later formed an influential coalition of environmental groups (Isar Alliance) that advocated for, and ultimately co-designed, the NBS. Indeed, the Isar Plan was in the vanguard of the participatory approach by actively engaging environmental NGOs, residents, and other stakeholders in the planning and, to some extent, the co-design of the NBS (Zingraff-Hamed et al. 2019; Sartori 2012).

### Wolong case

China’s Natural Forest Conservation Program (NFCP), which was initiated and financed at the national level, is probably the world’s largest NBS program in both spatial coverage and financial support with major *foci* on climate adaptation, biodiversity, and flood risk reduction (Liu et al. 2001). In the late 1990s, the NFCP was implemented in the Wolong Nature Reserve, China’s flagship protected area located in the Upper Yangtze River, a global hotspot region for both biodiversity and disasters (Viña et al. 2011). The program pursues its goals through a nationwide logging ban and large-scale afforestation and reforestation, using a “payment for ecosystem services” financial scheme (Chen et al. 2009). Success of the NFCP in Wolong can largely be attributed to innovations in the vision, planning, interventions, and changes in institutional frameworks. The policy intervention is composed of a “carrot and stick” forest management concession contractual system. The novel idea, which emerged from discussions at the village scale, was to pay households to monitor logging in the community forests, which made them part of the solution instead of part of the problem. Reportedly, this approach as implemented in the case area increased household income and welfare of local residents, although studies of the NFCP in other areas report mixed results (Yang et al. 2013).

### Nocera Inferiore case

In 2019, an NBS for mitigating landslide risk was finalized in the town of Nocera Inferiore in southern Italy. The NBS measures were fully financed by the national government and included maintenance and remediation of the mountain slope, channel lining, and provision of vegetated and stone gabions, all aimed at reducing erosion and landslide risk due to frequent rainfall events. The NBS is part of a more comprehensive and hybrid plan for forest and risk management that includes, for example, complementary grey infrastructure, the improvement of walking paths, and improved management of public and private forests.

The history of the project dates back to March 2005, when a landslide on one of the highest-risk areas of the town, the Mount Albino slope, caused three deaths and extensive property damage. Three years later, a €24.5 million risk mitigation project — consisting of mainly unsightly concrete (grey) measures — prepared by the Regional Emergency Commissariat was rejected by the Municipal Council with the support of many citizens and local associations. In the wake of the rejection, two Emergency Commissioners were appointed, and a €7.2 million budget was earmarked for a risk mitigation plan. The stalemate signaled the need for more inclusive and transparent policies and decision-making processes. The municipal authorities were hence keen to involve the residents of Nocera Inferiore in the preparation of a new landslide risk mitigation plan. The entry point to public participation was provided by a research project funded by the European Commission (EC) involving a 2-year co-design process structured as a series of workshops with a group of selected residents, experts, and several parallel activities open to the public (Linnerooth-Bayer et al. 2016; Scolobig et al. 2016). This eventually led to the implementation of the NBS in 2018–2019 (Martin et al. 2019).

## Emergent characteristics of transformative adaptation

In this section, we delve into the three NBS cases to explore if and how transformative visions, planning, interventions, and institutional frameworks (Fig. 1 and Table 1) emerged during the policy process.

### Transformative visions

As described in Table 1, a transformative vision is systemic and path-shifting; it recognizes the drivers or root causes of the risk; and it mobilizes leadership and advocacy coalitions. The Isar, Wolong, and Nocera case studies demonstrate the emergence of NBS visions with many or even most of these characteristics and which competed with conventional “grey” solutions in the policy arena, eventually gaining the support (often through a compromise) for a nature-based solution. It is important to emphasize, however, that a transformative vision emerged from policy processes that lasted over several years and that involved multiple conflicting advocacy groups at least in the Isar and Nocera Inferiore cases.

More precisely, the NBS projects implemented in the three case studies were the result of systemic visions regarding how to reduce extreme flood and landslide risk or adapt to climate change, be it through the far-reaching restoration of a river (Isar), first-of-its-kind innovations in community forest management (Wolong), or prioritizing NBS as a first defense against landslides through improved maintenance and remediation of the mountain slope (Nocera Inferiore). In Munich, the re-naturalization of the Isar River had systemic characteristics in that it reconciled competing stakeholder interests and worldviews: (i) flood risk reduction with grey measures, (ii) restoration of the ecology of the “wild river”; and (iii) enhanced recreational options with increased property values. Note that to some important extent these interests/worldviews conflicted, which called for a lengthy negotiation among the various advocacy coalitions. The NBS was not only systemic but also path-shifting because it stood in opposition to conventional practices that had been singly based on grey and passive measures. This was also the case in Nocera Inferiore. Members of environmental associations, some of whom were also members of the local municipal council, stressed the imperative of taking a more systemic, holistic, and ecological view of the mountain and its maintenance. Thus, the NBS that were implemented in the Isar and Nocera cases moved away from the beaten track, addressed competing interests and worldviews, and questioned traditional values by challenging the assumptions of the status quo.

Similarly in Wolong, a systemic solution emerged that challenged the *status quo*. The district government in an apparent accommodation with the national party authorities took the lead in a unique system of community-based monitoring of illegal logging, which emerged as a deviation from the earlier approach based on sanctions and signaled a major breakthrough in preserving the forest. A systemic and integrated vision emerged based on the improvement of forest management and flood and landslide risk reduction as explained by an interviewee working for the Department of Natural Resources Management:

Wolong, being also a special district, is unique in China’s protected areas. We are not only a reserve, but also a government. While conservation and pandas are always of highest priority for us, we had no choice but to find solutions that may help us address development and disaster issues in synergy with conservation…. Lucid waters and lush mountains are invaluable assets (Interviewee #17).

The policy intervention supporting this transformative vision complemented the traditional “sticks” approach of sanctions for illegal logging with “carrots” in the form of payments to household groups that were successful in preventing logging in the forest areas assigned to them. This intervention was path-breaking insofar as it shifted responsibility from the authorities to local residents, thus creating buy-in and ownership. This has proven successful in maintaining forest cover, which serves as an essential NBS for flood and landslide protection, biodiversity by assuring panda habitat, and ecotourism. At the same time, this intervention increased social inclusion and household incomes at least as reported in this case study area.

Turning to the third characteristic of a transformative vision, the case studies show the importance of creating a space for those advocating holistic NBS solutions. In the Isar and Nocera cases, advocacy coalitions were instrumental, even essential, in bringing forward transformative visions. In the Isar case, a coalition was formed that rallied many different environmental NGOs under the long-standing Isar Alliance (Sartori 2010). This coalition voiced its overarching vision to restore a more natural ecosystem by allowing the river to meander freely and regain a “wilderness character,” which proved key to paving the road for an NBS (Kangler et al. 2014; Binder et al. 2015). As noted by a member of the Alliance:

The members of the Isar Alliance stood up for the Isar restoration. This was picked up by the politicians. Munich’s mayor also supported this (Interviewee #6).

These groups acted as agents of change to bring forward a new vision for disaster risk reduction with NBS at its heart. In Nocera, not only the environmental associations but also many residents had been highly critical of past “grey” solutions for landslide risk reduction. They particularly questioned their aesthetic and environmental impacts, high construction and maintenance costs, and the necessary expropriation of private land (Chiavazzo et al. 2018). In the Isar case, there was a significant transition in the operating culture of the Munich Water Agency — from a focus on grey infrastructure for flood protection to a more holistic and nature-based approach. This innovative vision was brought about by, among other factors, a group of young and ecologically committed staff members who believed that flood protection could be achieved other than through the business-as-usual approach (in this case, raising the river embankments). In the words of a former member of the Munich Water Agency:

The “concrete faction” in the Water Agency had retreated. Young engineers and landscape planners were the ones in charge now (Interviewee #7).

In Wolong, the district government partner appeared as an advocate by managing to change the framing of the deforestation problem and solution. In this and the other cases, the initial minority voice had novel ways of framing the problems and proposing innovative solutions to address them. Importantly, they often mobilized human or economic resources to realize them.

Finally, although the visions in all three case studies include considerations of the root causes of the risk, little has been done to address those causes. In Nocera Inferiore, opposition to environmentally detrimental anthropogenic practices, such as road building, industrial activities, and even the location of power lines at the edge of the slope, are emphasized by multiple interviewees. In Munich, locals were aware of the various anthropogenic pressures that had shaped the Isar (e.g., channelization of the river and dams for electric power plants), and these, in part, catalyzed the new vision for a restored Isar. In Wolong, it had become evident that a root cause or driver of deforestation was the poverty in the area. However, in all three cases, the core problems were not directly addressed, and often remain a major battle for local NGOs (e.g., in the Isar case) (Bayerischer Kanu-Verband 2021).

The failure to address the systemic sources of flooding and landslide risks in all cases points to a fundamental problem in governance for sustainable solutions, notably the narrow and often siloed mandates of the administrative bodies in charge. One can reasonably ask why the Munich Water Agency should be responsible for the anthropogenic pressures that had led to river channelization, or for climate adaptation; why the focus of the government authorities in Wolong should go beyond the pressing deforestation problem; or why the relevant policy actors in Nocera Inferiore should be responsible for limiting development on fragile slopes. In each case, this responsibility goes beyond the mandates of the respective authorities, which signals the need for transformational institutional changes, for example, that expand mandates or that dictate inter-agency coordination, to encompass a broad sustainability vision in planning for major governmental infrastructure investments (more on this in the “Transformative institutional frameworks” section).

In conclusion, many characteristics of transformative visions were present in the case studies (Table 3). Interestingly, our findings also reveal that these visions were often the result of a long and time-consuming process of negotiation of distinct aspirations among those advocating for NBS. Indeed, the Isar case vision of a naturalized and “wild-flowing” river through an urban center as part and parcel of flood risk management was in the making for over two decades. Although less lengthy, the narrative of a naturalized and more holistic landslide intervention in Nocera Inferiore took several years to dominate the public agenda. In Wolong, adding “carrots” to the “sticks” in the control of illegal logging emerged only after a complex interactive co-generation process with the authorities and forest communities. Thus, the evolution of the transformative visions in the Isar, Nocera, and Wolong cases adds evidence to the hypothesis that transformation is not a “one-off” system change but a complex and nuanced process that evolves over time.

**Table 3:** Characteristics of transformative visions, planning, interventions, and institutional frameworks

Element	Characteristic	Isar	Wolong	Nocera
Transformative vision	Systemic	*	*	*
	Path shifting	*	*	*
	Mobilizes advocacy coalitions	*	*	*
	Addresses root causes or drivers			
Transformative planning	Inclusive	*	*	*
	Co-production	*	*	*
	Equitable	*	*	*
	Based on open data systems			
Transformative interventions	Scalable		*	
	Sustainable in the long term	*	*	*
	Future-oriented	*	*	*
Transformative institutional frameworks	Polycentric	*	*	*
	Promoting social justice			
	Catalytic			

### Transformative planning

The four key characteristics of transformative planning are inclusivity, equity, co-production, and open data (see Table 1). In each of the three cases, considerations about inclusivity and co-production/co-design were important in the NBS planning stages, but also importantly different in their extent.

In Nocera Inferiore, a novel participatory process including residents and experts was carried out to co-design a risk reduction plan that proved decisive in unblocking a local policy stalemate on the landslide issue. In the Isar case, an ad hoc yet inclusive participatory process emerged that shaped the outcome toward an NBS with stakeholder input included in the final design by the landscape architects. Even if a formal evaluation of this extensive stakeholder engagement process has not been conducted, its importance for the Isar Plan’s success was noted by several stakeholders (Martin et al. 2019; Lupp et al. 2020), including:

We did not have any clear rules or guidelines for stakeholder involvement…. But I think it was very important for the success of the project that a kind of participation and stakeholder involvement was continuously established or, in other words, that a change in culture was developed. In the end this is the only way to realize such large projects (Interviewee #2)

In China, an almost unprecedented procedure of household consultation was carried out by the Chinese authorities. Moreover, in 2019, China announced the Natural Forest Conservation and Restoration Policy (NFCP) Plan (CCCPC and SC 2019). The role of public participation and co-production of NFCP in the Wolong Nature Reserve (WNR), which dated back almost 20 years, was clearly echoed in Article 20 of the Plan:

Natural forest conservation should be a long-term, multi-generation effort with strong public participation, co-production and benefit sharing. Non-structural measures, such as formulating locally adapted rules … should be encouraged in order to cultivate new ecological ethics and behavior norms for sustainable forest management….

Equity also emerged as a consideration in all three cases. In the Nocera process, by ensuring the safety of the residents in the slope area, the NBS increased equity in risk distribution at the municipal level, especially between residents of the town versus those living on the mountain slope (Interviewee #40). As reported by a member of a local environmental NGO:

There is a need to guarantee equal safety standards for all families living in Nocera and on the Mount Albino slope. We should ideally have a risk map with the same color (for risk level) everywhere, but I am not sure this is technically feasible.. (Interviewee #37)

Equity emerged differently in Munich, where an important narrative on a restored Isar as accessible to all — bringing together people from all walks of life, cultures, backgrounds, and generations — took place. It was also highlighted that having a recreational space at the heart of the city allowed wider access by public transport, a privilege otherwise reserved for people owning a car. This was highlighted by a member of a local council:

The biggest success was that people got a river they can use in a city of millions. Although ecological aims were fulfilled, the social success, i.e., the restored accessibility of the river for all people, surpasses that. (Interviewee #6)

In Wolong, the idea of paying households to prevent illegal logging, instead of sanctioning them for allowing or taking part in illegal activities, was a powerful mechanism for motivating households in one of China’s poorest regions. Indeed, payments for this ecosystem service were a significant addition to household income and financial security. The transition from “sticks” to “carrots” for financing the conservation of common goods was considered by many interviewees as a more equitable forest management strategy.

Open data systems were not in evidence and perhaps less relevant in the cases studied. In Isar and Wolong, this could be due to data systems and technologies being less developed at the time of the NBS implementation or to data being less easily and readily shared. Nevertheless, the Isar and Nocera cases were characterized by extensive communication strategies during the NBS implementation phases (e.g., guided tours, an information point, public lectures and presentations, educational trails, and information boards).

Importantly, we find further evidence in the planning procedures that transformation is not a “one-off” system change but a complex and nuanced process that evolves over time. In none of the cases was an inclusive participatory co-design process in place at the start, nor was participation viewed as having an influence on the final outcome. To the contrary, in each case, the participatory elements of the NBS process evolved (in Nocera, a co-production process emerged only as a result of an EU Horizon 2020 project). The emergence of actors outside of the decision authorities and the receptiveness of the authorities were crucial for the resulting NBS.

### Transformative interventions

Turning to the case outcomes, the NBS projects implemented in Munich, Nocera Inferiore, and the Wolong nature reserve all meet two key characteristics of transformative interventions, namely, “sustainability” and “orientation to the future.”

The Isar intervention is considered transformative because it successfully turned a formerly concrete and unsafe riverbank into a green/blue recreational space, which has become an important emblem of this city of millions. Sustainability may not have been explicitly at the core of the planning and implementation measures; however, with its focus on the dimensions of environmental restoration, recreation, and flood protection, the Isar Plan fulfilled both the social and ecological dimensions of sustainability despite that economic sustainability (in terms of economic development and benefits) was little referred to in the interviews. Economically speaking, the Isar Plan is known to have cost more than an alternative grey solution. It has also incurred high maintenance costs, although these have never been compared empirically with a hypothetical structural solution (Wetzel 2016). While flood protection is generally viewed as the principal benefit of the project and equally as the rationale for financing its costs (approximately €35 million), the Isar Plan’s long-term and future-oriented co-benefits (ecological restoration and recreation) are widely portrayed and perceived as the project’s major success, as reflected in stakeholder interviews:

Good environmental status [under the Water Framework Directive] also encompasses social function. If I aim for a good status, this must serve not only nature, but also humans. (Interviewee #5)

It should be noted, however, that over a decade has passed since the initiation of the project, allowing co-benefits to develop and flourish. Thus, from a retrospective standpoint, the future orientation of the project may seem to have emerged more strongly.

Sustainability was also key in Nocera, especially given that the landslide NBS was viewed as part of a more comprehensive plan that included, for example, the creation of a natural park at the foot of Mount Albino, the improvement of walking paths, small-scale organic farming, better management of public and private forests, and also some structural/grey measures — all benefits in the here and now. The view that these green measures could not sufficiently mitigate the landslide risk led some actors to advocate for a green-grey hybrid approach. As reported by a municipal technical officer:

We built an NBS because of the limited funding available. Moreover, we believe that it has a low environmental impact. Yet, to mitigate the risk on the entire slope, more funding is necessary and, most likely, some structural risk mitigation measures will have to be built in the future. (Interviewee #27)

While risk mitigation appears to be the core focus, sustainability was the core mission of several NGOs (e.g., the Montagna Amica victims committee in Nocera) that supported the NBS-oriented vision for risk reduction.

Conserving forests in Wolong is undoubtedly a sustainable and future-oriented endeavor. Yet, arguments for benefits in the here and now including, for instance, maintaining panda eco-tourism and prevention of downstream flooding, dominated the NBS rationale.

Except for the Wolong case, the scaling of the NBS to wider geographic regions or the creation of enabling conditions for its wider application was not part of the policy dialogue. The NFCP consisted of a nationwide logging ban and large-scale afforestation and reforestation policy, which, as discussed above, involved financial incentives for community-based monitoring of illegal logging. As reported by an interviewee working at the China Conservation and Research Center for the Giant Panda:

Illegal logging is a national and provincial level problem. Wolong alone cannot solve it unless the surrounding areas all work together in enforcement of checking, confiscating, and punishing illegal logging, including the timber market. After NFCP, the legal and transaction cost of illegal logging increased substantially in Sichuan. This also indirectly helped reduce deforestation pressure in Wolong. (Interviewee #27)

Long-term plans to scale NBS adoption were not evident in the interviews nor in the case study analyses of the Nocera Inferiore and the Isar cases. There are many reasons for this. In Italy, there was an absence of regional or national regulatory frameworks to support the wide-scale uptake of NBS, limited funding available to support scaling, and opposition from stakeholder groups (e.g., those strongly supporting the implementation of grey measures). Likewise, while the Isar case is known to have inspired other river restoration projects in Germany and beyond (Martin et al. 2019), scaling was not voiced as an intent by any of the policy actors.

Similar to the earlier discussion on the absence of addressing “root causes,” one can again reasonably ask why the responsible authorities should, indeed, be concerned with scaling NBS to other regions or to other sectors outside of their jurisdiction. However, the agents (especially in the Isar and Nocera cases) extended beyond that of the responsible administration. It is notable, for instance, that the Munich Water Agency was mandated to include ecological and social concerns in its plans for flood protection (paving the way for the Isar Plan), but it did not have the mandate or financing to intervene in rivers beyond its jurisdiction. Yet, agents in the Isar policy process extended beyond the municipal water authorities, for example, including the Bavarian water authority and other state agents, which could have built on the Isar case to institute the necessary institutional reforms (e.g., the polycentric working group) to enable change more broadly. Likewise, landside mitigation in Nocera Inferiore involved agents reaching far beyond the municipality, including the regional, river basin, and national authorities; yet, there was little institutional reform to enable landslide NBS beyond the Nocera case.

A further insight from the case studies is the importance of co-benefits for transformative interventions, ranging from ecological resilience, economic growth, and recreation to health. The NBS implemented in each case presented substantial co-benefits, reaching beyond climate adaptation and disaster risk reduction, which added significantly to their rationale, appeal, and eventual realization. Although the values of these co-benefits — particularly social values — often remain unaccounted for in formal cost–benefit analyses, they represent a crucial element for several characteristics of transformation, such as catalyzation and future-orientation.

### Transformative institutional frameworks

As described in Table 1, the literature highlights three key characteristics of transformative institutional frameworks: (i) promotion of multi-scale and cross-sectoral polycentric arrangements; (ii) promotion of social justice; and (iii) capacity to promote deliberate shifts to trigger cascading impacts within structures that are beyond the institution’s direct mandate or reach.

The case studies showcase the emergence of transformative institutional frameworks in the form of polycentric arrangements, participatory processes, and new instruments for incentivizing behavior, all of which proved instrumental for NBS design, planning, and implementation (Table 1). Particularly critical were polycentric arrangements that cut across organizational responsibilities and sectors to include NBS attributes beyond disaster risk reduction, such as nature and biodiversity protection (Isar Plan, Nocera, and Wolong), urban planning, water quality, and waste management (Isar) and landscape planning, tourism, and household economic security (Wolong). In all cases, the multi-scale and cross-sectoral collaboration (key characteristics of polycentric governance) broke the administrative silos so typical of public administrations.

In the Isar case, this collaboration was initiated by ecologically committed staff of the municipal government and the local water authority, who formed a multidisciplinary working group that was unprecedented for flood management (Zingraff-Hamed et al. 2019). This governance arrangement proved key to enabling the successful implementation of the Isar-Plan: the so-called Isar-Plan Working Group, created as an informal working group in 1987 and institutionalized by Parliament in 1995. This working group marked a critical milestone in the Isar story, as it dispersed decision authority across multiple organizations and authorities that went beyond just flood protection. One of the group’s aims was to discuss and resolve conflicts before they could escalate.

The Isar Plan Working Group served to discuss challenges amongst various experts…. We said we will develop what we want to build in Munich together. This was the first time that such a Working Group had been created. (Interviewee #2)

In Wolong, an equally unprecedented collaboration developed across the national, provincial, and local scales, each with different agendas spanning disaster risk reduction, conservation, and economic wellbeing. This collaboration was catalyzed in large part by a cross-departmental committee led by two strong NBS advocates.

In Nocera Inferiore, the NBS agenda became part of a much broader agenda that brought together multiple sectors across administrative scales and jurisdictions. As reported by one technical officer:

Waste management, urban development, risk reduction are all part of a broad environmental agenda. This also reflects the environmental awareness which changed over time. Thanks to a coalition of local politicians, officers and consultants, we have been able to push forward a new environmental agenda. (Interviewee #47)

At the same time, the interviews reveal a lack of overt attention to social justice or any explicit intentions to address the causes of injustice and power imbalances that are present in all the case studies, albeit to varying extents. Indeed (procedural and distributional), social justice considerations were not explicitly addressed or included in the NBS design, planning, or implementation. This does not mean that the NBS, themselves, did not promote equity: the Isar River became a recreation area for all residents; the ecoservice payments in Wolong were a significant addition to low household incomes; and the Nocera landslide mitigation reduced risk to vulnerable communities. Moreover, the creation of coalition groups, particularly in the Nocera and Isar cases, represents non-institutionalized attempts to give like-minded citizens a voice prior to and during the NBS implementation, where individuals would otherwise not have been heard. Yet, claims of power redistribution were only seldom part of the justification for the NBS.

The interviews also disclosed limited evidence on intentions to catalyze a broader recognition of the benefits of NBS or their scaling. There were no agency-led shifts (e.g., adoption of a tool, model, or framework) to trigger cascading impacts within governance structures that are beyond the governing institution’s direct mandate or reach. For instance, the Isar Working Group remains a unique institutional arrangement with no (formal) cascading effects on formalized administrative procedures or German legislation. The results highlight the difficulties inherent in upscaling NBS beyond their initial institutional scope and scale. Indeed, while the selected cases might have inspired future NBS projects, they did not catalyze formal changes in local or national legislation and policy.

Most noteworthy for transformation in the NBS-enabling governance frameworks are the ad hoc polycentric arrangements that evolved during the policy process in all three NBS cases, providing evidence that transformation is an evolving and nuanced process. However, these cross-sector and cross-scale processes to internalize the full range of NBS benefits within the planning, instrumentalization, and implementation of NBS have not been permanently institutionalized. For example, the Isar Working Group, along with the Nocera coalition of local advocates and the Wolong cross-departmental NFCP committee, were put into place in an ad hoc fashion with no subsequent replications or legislation to ensure their permanency.

## Discussion

In this discussion, we draw attention to the processes of design, planning, and implementation of NBS projects. The small sample of three case studies, along with their diversity in political, financial, socioeconomic, and ecological terms, precludes the creation of generalizable comparisons for universal transformative adaptation practices using nature-based solutions (Yin 2014; Martin et al. 2019). We thereby aim to relate concepts of transformation to practice (thus attempting to fill the operational gap of transformation) and to provide key insights acknowledged by the interviewees.

### Transformative visions, planning, institutional frameworks, and interventions

This study shows that none of the cases, the Isar-Plan, Nocera, or Wolong, fulfills all the characteristics identified in the literature as being key to obtaining transformative gains. However, transformative visions, planning, and interventions emerged throughout the NBS policy deliberations, and some characteristics are present in all the NBS projects under study (Table 3).

For example, all visions supporting the realization of the NBS projects adopted a systemic approach initially brought forward by minority/pressure groups that were subsequently able to mobilize human and economic resources or influence political and technical decisions. Indeed, in each case, a transformative vision emerged and eventually dominated the planning and intervention discourse: the “wild-flowing” river in Munich; natural engineering measures in Nocera, and moving from “sticks” to “carrots” in Wolong. A major result of the analysis is recognition of the emergent character of transformative change.

Planning was generally inclusive and co-designed with a wide array of stakeholders. It is noteworthy that equity was not an overt consideration in any of the three cases; yet, it emerged, linked either to access to nature for all (e.g., Isar case) or as an important consideration in risk distribution (e.g., Nocera). Procedural equity in the form of inclusive participation was also a “silent” or unplanned characteristic of the cases. In no case was an inclusive participatory co-design process in place at the start, but it evolved — often ad hoc — over the planning process.

Interventions were generally future-oriented and sustainable; yet, the scaling of NBS interventions was difficult to achieve in practice because of regulatory, institutional, and financial barriers. Challenges to scaling NBS into policy agendas have been widely recognized as a major barrier for mainstreaming NBS (e.g.,Calliari et al. 2019; Cohen-Shacham et al. 2019; Frantzeskaki 2019; Scolobig et al. 2020). Importantly, recognition of NBS co-benefits beyond disaster risk reduction was essential for their rationale, appeal, and eventual realization, despite the absence of formal methods, like cost–benefit analysis, to capture their social, economic, ecological, and other benefits (Josephs and Humphries 2018).

Our analysis reveals that a main gap in achieving transformative adaptation for publicly financed NBS has been creating transformative institutional frameworks rather than transformative visions, planning, or interventions (see the “Transformative institutional frameworks” section). For some, the notion of a transformative institution might suggest an oxymoron, juxtaposing fundamental change with stability; yet if transformation is seen as an emergent process, with gains and losses, then institutional processes, memory, and learning become key components in shaping transformation over time. Most noteworthy are the ad hoc polycentric arrangements that evolved during the policy process in each NBS case, again providing evidence that transformation is not an abrupt systemic change but part of an evolving complex process. Although the cases showed institutional commonalities in collaborations among the authorities, all successful institutional arrangements were short-term and dependent on the motivation of local champions. For example, the multi-scale (Isar and Wolong) and cross-sectoral (Nocera and Isar) collaboration (two characteristics of polycentric governance) broke administrative silos that are typical in public administrations. Analogous to the Isar working group, a cross-department NFCP committee emerged in the Wolong case, led by two governmental champions with rich local knowledge, and this bridged across separate disaster-protection-conservation-development agendas. However, these collaborations were primarily ad hoc and short term. Also, our results reveal a lack of overt attention to social justice and promotion of catalytic change, two key characteristics of transformative institutional frameworks.

Other studies share the same finding (e.g., Frantzeskaki et al. 2020) and highlight institutional spaces that enable collaborative learning, including, for example, large-scale research programs such as those funded by the European Union’s Horizon program — but are unfortunately limited in time and funding. As a result, these spaces leave transformative adaptation largely dependent on the engagement, skills, and motivation of local champions, change/innovation managers, or policy entrepreneurs.

Our results suggest the need for establishing more permanent institutional frameworks that are adaptive, multi-scale, cross-sectoral, and also well enough established to guarantee the delivery of transformation on a permanent basis. It appears that long-term and permanent solutions are needed to promote new institutional settings that are better able to deliver transformation. An option is the creation of new institutions devoted to adaptation and NBS promotion with their own budgets and a clear political mandate (Runhaar et al. 2018). An example could be the establishment of climate offices or secretariats to assist agencies in the implementation of climate strategies. Successful examples are provided, for example, in Braunschweiger and Pütz (2021). Another option for promoting change is the integration of transformative adaptation goals into various sectors and policies, often called mainstreaming (Braunschweiger and Pütz 2021). Wamsler and Pauleit (2016) identify six different mainstreaming strategies including add-on, programmatic, managerial, intra- and inter-organizational, directed, and regulatory strategies. Other catalysts of institutional innovation include new regulations or climate policy updates (where they exist), strong political will and commitment, wider capacities, setting of cross-competing priorities, cross-sectoral cooperation formal mechanisms, and/or integrated planning.

### Limitations

This analysis has adopted a framework that enables a more action-oriented analysis of gaps in transformative adaptation through NBS. It draws on existing literature to evaluate the transformative potential of government-led adaptation projects at a local or subnational government level, and tests it through application to three empirical cases. Robust testing of this framework will certainly need further iterations. Indeed, some methodological considerations related to the analysis deserve attention. Our methodology builds exclusively on qualitative evidence, collected through extensive desktop research and interviews. Quantitative evidence, including, for example, indicators for monitoring changes in transformation characteristics over time, would probably strengthen the robustness of the framework. Future research should focus on the development of more structured and mixed qualitative/quantitative monitoring, evaluation, and learning (MEL) approaches that can help better assist future transformative adaptation projects (e.g., toolkits or composite indices presented in EEA (2015, 2020); Lesnikowski et al. (2015); UN Environment DTU Partnership (2017); ETC/CCA (2018); and Hallegatte et al. (2020). Yet, MEL approaches for transformative adaptation are unlikely to succeed by themselves as a narrow quantitative assessment tool, but more so as part of a reflexive and iterative framework. Such MEL frameworks can provide an evidence-based pathway through unfolding adaptation in which technological, social, and nature-based adaptations interact — in planned or unplanned ways — and across identified transformative adaptation actions.

Moreover, the paper captures transformative aspects of planning and implementation of projects, but since the analysis focuses on the policy processes and implementation rather than on a representative assessment of beneficiaries’ opinions about transformative adaptation, it does not capture transformative outcomes.

While carrying out research across multiple countries presents challenges for comparative analysis, we believe that such a systemic approach is critical for future robust testing of the framework and its potential to influence practice (Frantzeskaki et al. 2020). Our case studies provide partial evidence on transformation in the context of administrative governance. They do not, for example, address market mechanisms for financing and implementing NBS because all three projects were financed by public budgets. Since the political processes leading to the project financing were not available to us, political governance (in contrast to administrative governance) was not at the core of the analysis. Still, it is critical to acknowledge the different levels of financing in the three case studies, which affects how governments are able to address transformative adaptation potential.

More generally, the availability, collection, and analysis of data related to case studies on transformative adaptation raise questions concerning how to frame the data in terms of choice, availability, and the skills and tools needed to collect and analyze them.

Lastly, the range of interpretations of transformative adaptation currently in circulation calls for a continuing, rigorous, and structured review of practices to present experiential findings. Open access tracking using qualitative and quantitative tools are still lacking. There are limited comparative analyses that can present experience in conjunction with the range of defining characteristics of transformative adaptation (see also Palomo et al. 2021). These analyses will allow to better understand, e.g., transformative adaptation dynamics through NBS projects in contrast with other types of adaptation projects. This research can also allow to better characterize how transformative adaptation differs from other types of adaptation.

## Conclusion

The need for transformation in climate change adaptation is now firmly accepted and can claim normative orientation through numerous international agreements, including the UN sustainable development goals. Transformation has thus become part of the policy language. It is invoked in multiple contexts, including most currently in reference to recovery from COVID-19 (Deubelli and Mechler 2020; Roberts and Pelling 2020).

This paper has offered a practical perspective on the transformation literature by exploring the conceptual framing from the perspective of real-world experiences with regard to the implementation of major nature-based solutions in Europe and China. Specifically, the paper proposes a framework for assessing transformative change and tests its usefulness in terms of identifying the strengths and weaknesses of processes intended to support transformation toward more sustainable and equitable societies. While the scope has been limited to administrative governance, excluding the political processes that had led to financing the NBS, the framework provided a basis for critical discussion of how the vision, planning, interventions, and institutional frameworks implemented in the selected case studies can be considered as transformative. The case analysis in Munich (Germany), Nocera Inferiore (Italy), and Wolong (China) adds evidence to the view that transformation is a dynamic process and that its key elements evolve over time.

The conceptual framework also proved useful in uncovering the strengths and weaknesses of the procedures and outcomes in the case studies. It showed, for instance, that inclusive co-design planning processes and novel polycentric governance institutions can emerge from a process initially dominated by government authorities. Importantly, the framework also revealed those aspects of the NBS processes that were not fully transformative. Indeed, in none of the cases were all the characteristics of transformative adaptation met. The stakeholder narratives and ultimate interventions did not, for instance, fully address the root causes of the problems, which arguably stem from systems steeped in historical neglect of both the environment and social equity. Moreover, and importantly, the cases revealed little intent to scale the NBS, for instance, through duplication, enabling legislation and more permanent institutions, which is perhaps the most revealing result of the analysis.

Indeed, our results reveal that strong political will is needed to deliver long-term and permanent transformation. The cases highlighted options for strengthening capacities to deliver transformation, including the establishment of cross-competing priorities, cross-sectoral and multi-level integrated planning, new dedicated institutions, programmatic and regulatory mainstreaming.

Building on these results, the framework can assist practitioners, policymakers, civil society, and researchers to understand transformative adaptation and, importantly, to encourage it through systemic changes. Finally, it can help ensure that societal and systemic implications of specific transformations are better understood from the outset.

## Supplementary Information

Below is the link to the electronic supplementary material.

Supplementary file1 (DOCX 18 kb)

## Data Availability

The datasets generated and/or analysed during the current study are not publicly available due to privacy considerations. Anonymous interview transcripts (respectively in German, Italian and Chinese) are available from the corresponding author on request. The interviewee list, organisation/affiliation, method and data of interview are available in the supplementary information.

## Declarations

### Disclaimer

The funders had no role in the design of the study; in the collection, analyses, or interpretation of data; in the writing of the manuscript; or in the decision to publish the results.
