# Supplementary material for: Transformative adaptation through nature-based solutions: a comparative case study analysis in China, Italy, and Germany
Source: Reg Environ Change. 2023 May 2;23(2):69. doi: 10.1007/s10113-023-02066-7 (PMC10152420; doi:10.1007/s10113-023-02066-7)
Supplement: Supplementary file 1 — Supplementary file1 (DOCX 18 kb) [file 10113_2023_2066_MOESM1_ESM.docx]

**Annex: Interviewee list and interview details**

| Interviewee number | Case study site | Organization or affiliation | Interview method | Date of interview | Interviewer |
| --- | --- | --- | --- | --- | --- |
| 1 | Isar case | City of Munich Planning Division | Face-to-face | 18.03.2019 | J.M. |
| 2 | Isar case | Munich Water Agency | Face-to-face | 19.03.2019 | J.M. |
| 3 | Isar case | Burkhardt Engelmayer Landscape Architects | Face-to-face | 19.03.2019 | J.M. |
| 4 | Isar case | Isar Alliance / Mühltal initiative | Face-to-face | 20.03.2019 | J.M. |
| 5 | Isar case | Münchner Forum | Face-to-face | 20.03.2019 | J.M. |
| 6 | Isar case | NGO Save the Isar now! / Isar Alliance | Written | 10.04.2019 | J.M. |
| 7 | Isar case | Canoe Association/ Isar Alliance | Telephone | 04.04.2019 | J.M. |
| 8 | Isar case | City of Munich Construction Division | Telephone | 22.08.2019 | J.M. |
| 9 | Isar case | Bavarian Fisheries Association | Telephone | 26.06.2019 | J.M. |
| 10 | Isar case | Bavarian Ministry of the Environment | Telephone | 11.07.2020 | J.M. |
| 11 | Isar case | Isar Alliance | Written | 08.08.2019 | J.M. |
| 12 | Isar case | Isar Valley Association | Telephone | 01.08.2019 | J.M. |
| 13 | Isar case | Journalist and author | Telephone | 08.08.2019 | J.M. |
| 14 | Isar case | Technical University of Munich | Telephone | 01.03.2019 | J.M. |
| 15 | Isar case | Munich City Utilities | Telephone | 28.08.2019 | J.M. |
| 16 | Wolong case | Wolong Nature Reserve Administrative Bureau Deputy | Telephone | 19.07.2019 | W.L. |
| 17 | Wolong case | Department of Natural Resources Management | Telephone | 18.07.2019 | W.L. |
| 18 | Wolong case | Department of Natural Resources Management | Telephone | 19.07.2019 | W.L. |
| 19 | Wolong case | Department of Economic Development | Telephone | 18.07.2019 | W.L. |
| 20 | Wolong case | Department of Social Development | Telephone | 15.09.2019 | W.L. |
| 21 | Wolong case | Administrative Office | Telephone | 15.09.2019 | W.L. |
| 22 | Wolong case | Wolong township local resident | Telephone | 20.07.2019 | W.L. |
| 23 | Wolong case | Wolong township local resident | Telephone | 20.07.2019 | W.L. |
| 24 | Wolong case | China Conservation and Research Center for the Giant Panda | Telephone | 20.07.2019 | W.L. |
| 25 | Wolong case | Peking University | Telephone | 20.09.2019 | W.L. |
| 26 | Wolong case | Michigan State University | Telephone | 20.09.2019 | W.L. |
| 27 | Nocera case | Municipal technical office | Telephone | April-September 2019 | A.S. |
| 28 | Nocera case | River Basin Authority (Autorità di Bacino Distrettuale Appennino Settentrionale) | Telephone | April-September 2019 | A.S. |
| 29 | Nocera case | Regional Coastal Ecosystem & Water Cycle Management Authority | Telephone | April-September 2019 | A.S. |
| 30 | Nocera case | International Center on Environmental Monitoring | Telephone | April-September 2019 | A.S. |
| 31 | Nocera case | University of Salerno | Telephone | April-September 2019 | A.S. |
| 32 | Nocera case | Municipal Civil Protection | Telephone | April-September 2019 | A.S. |
| 33 | Nocera case | Municipal Urban Planning Office | Telephone | April-September 2019 | A.S. |
| 34 | Nocera case | Regional Sustainable Education and Citizen Participation Office | Telephone | April-September 2019 | A.S. |
| 35 | Nocera case | Regional Environmental Agency | Telephone | April-September 2019 | A.S. |
| 36 | Nocera case | National Civil Protection | Telephone | April-September 2019 | A.S. |
| 37 | Nocera case | Environmental NGO (Montagna Amica) | Telephone | April-September 2019 | A.S. |
| 38 | Nocera case | Environmental NGO (Leonia) and municipal council | Telephone | April-September 2019 | A.S. |
| 39 | Nocera case | Civil society, resident in landslide risk area/participant in the process | Telephone | April-September 2019 | A.S. |
| 40 | Nocera case | Victims' committee | Face-to-face | June 2010- October 2011 | A.S. |
| 41 | Nocera case | Italian Environment Ministry | Face-to-face | June 2010- October 2011 | A.S. |
| 42 | Nocera case | Regional Agency | Face-to-face | June 2010- October 2011 | A.S. |
| 43 | Nocera case | Participatory process scientific advisor | Face-to-face | June 2010- October 2011 | A.S. |
| 44 | Nocera case | Emergency Commission | Face-to-face | June 2010- October 2011 | A.S. |
| 45 | Nocera case | Civil society, farmer living on the Mount Albino slope/ participant in the process | Face-to-face | June 2010- October 2011 | A.S. |
| 46 | Nocera case | Centre for GeoTechnologies, University of Siena | Telephone | April-September 2019 | A.S. |
| 47 | Nocera case | Municipal technical office | Telephone | April-September 2019 | A.S. |
